# Editorial: Regulation of immune cell trafficking in autoimmune diseases

**DOI:** 10.3389/fimmu.2024.1380258

**Published:** 2024-02-13

**Authors:** Wei Lin

**Affiliations:** ^1^ Department of Public Scientific Research Platform, School of Clinical and Basic Medicine & Institute of Basic Medicine, Shandong First Medical University & Shandong Academy of Medical Sciences, Jinan, China; ^2^ Department of Critical-care Medicine, Shandong Provincial Hospital Affiliated to Shandong First Medical University, Jinan, China; ^3^ Shandong Key Laboratory of Rheumatic Disease and Translational Medicine, Shandong Medicine and Health Key Laboratory of Rheumatism, The First Affiliated Hospital of Shandong First Medical University, Jinan, China

**Keywords:** immune cell trafficking, T cells, monocytes, signaling pathway, intravital molecular imaging

The migration of immune cells plays a vital role in the immune system’s development and surveillance. Immune cells become active and travel between tissues and organs to share information signaling through adhesion, chemotaxis, and ligand recognition. These mechanisms not only help the immune system eliminate harmful external factors but also stimulate immune cell activation and differentiation or generate immune memory (1). However, excessive and abnormal immune responses can lead to diseases in certain situations. Therefore, understanding the mechanisms of immune cell migration is essential for maintaining a balanced immune system and controlling diseases. In this Research Topic, we gather the reviews and original research articles discussing the latest insights into immune cell migration in various physiological and pathological contexts, emphasizing technological advancements in detecting immune cell migration.

Recruited T cells at the inflammatory site are crucial in causing tissue damage in various diseases (2). During infections, T cells are activated and migrate to the inflammatory site. Schmidt et al. reported that the accumulation of CD4+ T cells in the intestine was associated with intestinal homing receptors, namely α4β7 integrin, CCR9, and GPR15, during *Citrobacter rodentium and Strongyloides ratti* infections. Furthermore, IRF4 was demonstrated to be necessary for generating Th2 and Th17 cells and for effectively recruiting T cells to the intestine and the primary infection site by regulating the expression of α4β7 integrin, CCR9, and GPR15. The loss of one Irf4 allele already results in impaired responses. Thus, IRF4 is essential not only for differentiation but also for the migration of T cells.

During an infection, not only are antigen-specific T cells activated, but antigen-nonspecific T cells also play a dual role. They contribute to virus control and, at the same time, can lead to immunopathologies. Westmeier et al. discovered that MIF promotes its receptors on various subpopulations of T cells, specifically antigen-nonspecific T cells, which exhibit high cytotoxicity and proliferation. This suggests that increased MIF signaling may act as a regulatory mechanism for activating bystander T cells, potentially contributing to disease progression and severity.

γδ T cells, a subset of T cells expressing a unique TCR, are relatively less common than αβ T cells but are abundant in the gut mucosa (3). They are also found in the thymus, peripheral lymphoid tissue, and peritoneum. Unlike αβ T cells, γδ T cells can recognize certain bacterial, lipid, and tumor antigens bound to MHC Class I without the need for antigen processing. Additionally, γδ T cells are highly migratory and continuously interact with the epithelial cell layer and lamina propria cells. This migratory behavior is associated with maintaining small intestine homeostasis, controlling bacterial and parasitic infections, and responding to epithelial shedding induced by LPS. Mart´ınez-Vargas et al. have demonstrated that Myo1f plays a role in the adhesion and migration of γδ T intraepithelial lymphocytes. Myo1f, known for regulating filopodia dynamics and increasing adhesion and migration in neutrophils and B cells (4, 5), was shown in this study to be involved in the polarization of chemokine receptors and integrins, including CCR9 and α4β7. This polarization, impacted by Myo1f deficiency, results in reduced tyrosine phosphorylation, potentially affecting signal transduction. Thus, Myo1f regulates adhesion and migration in γδ T intraepithelial lymphocytes by mediating lipid raft-dependent CCR9 and α4β7 polarization, which can influence cell signaling.

The presence of monocytes infiltrating tissues is closely associated with the onset and progression of various diseases. Malmhäll-Bah et al.‘s research indicated that *CDC42* ^hi^CD14^+^ cells carried a metabolic signature (MetSig) including *ATP5BP, COX7A2, PSMB6, PSME3, GTF3C6, and GTF2E2*, which individually correlated to CDC42, and together identified the patients where clinical rheumatoid arthritis activity was dependent on the CDC42-related MetSig of CD14^+^ cells. The migratory phenotype of *CDC42* ^hi^CD14^+^ cells also reflected the functional importance of these cells in the Rho-GTPase-dependent inflammation in rheumatoid arthritis. These proteasome-dependent proteins may modulate the metabolic signaling and Rho-GTPase-dependent inflammatory signaling pathway in CD14^+^ cells and further promote T-cell expansion and invasion. Thus, metabolic signaling provides energy for cell migration and is also an important factor in intervening in cell migration.

Numerous advanced technologies and methods have been employed in the study of immune cell movement and trafficking and provide functional and visual evidence to uncover the mechanisms behind immune cell recruitment. The surveillance function of immune cells involves both long-range patrolling throughout the body and local scanning of cells and molecular signals in tissues. To comprehensively depict the essence of the immune response, it is vital to visualize the structure, distribution, interactions, functions, and dynamic changes of molecules and cells. Research instruments with high spatio-temporal resolution, extended dynamic monitoring capabilities, multi-parallel functionality, and 4D detection are essential (6). Intravital molecular imaging is considered the most suitable technique for monitoring the mobility and function of various immune molecules and cells in the tumor microenvironment (7–9). In this Research Topic, Peng et al. provide a concise overview of the intravital imaging workflow, multi-color labeling strategies, imaging windows, and methods for analyzing intravital imaging data based on their previous research. This review guides researchers on how to apply intravital imaging technology to their studies and gain a deeper understanding of the roles played by immune cells in cancer immunotherapy.

This Research Topic creates a platform for advancing research on the mechanisms of immune cell migration in the diseases. This topic discusses the role of IRF4, MIF, and myolf that could affect T cell migration in different subsets of T cells, as well as the metabolic signaling-related genes that affect CDC42^hi^CD14^+^monocytes to promote T cell expansion and invasion (**Figure 1**). These studies would explore novel mechanisms and therapeutic targets for these diseases, and the advanced technologies would provide more information for tracking immune cells in the disease.

**Figure 1:**
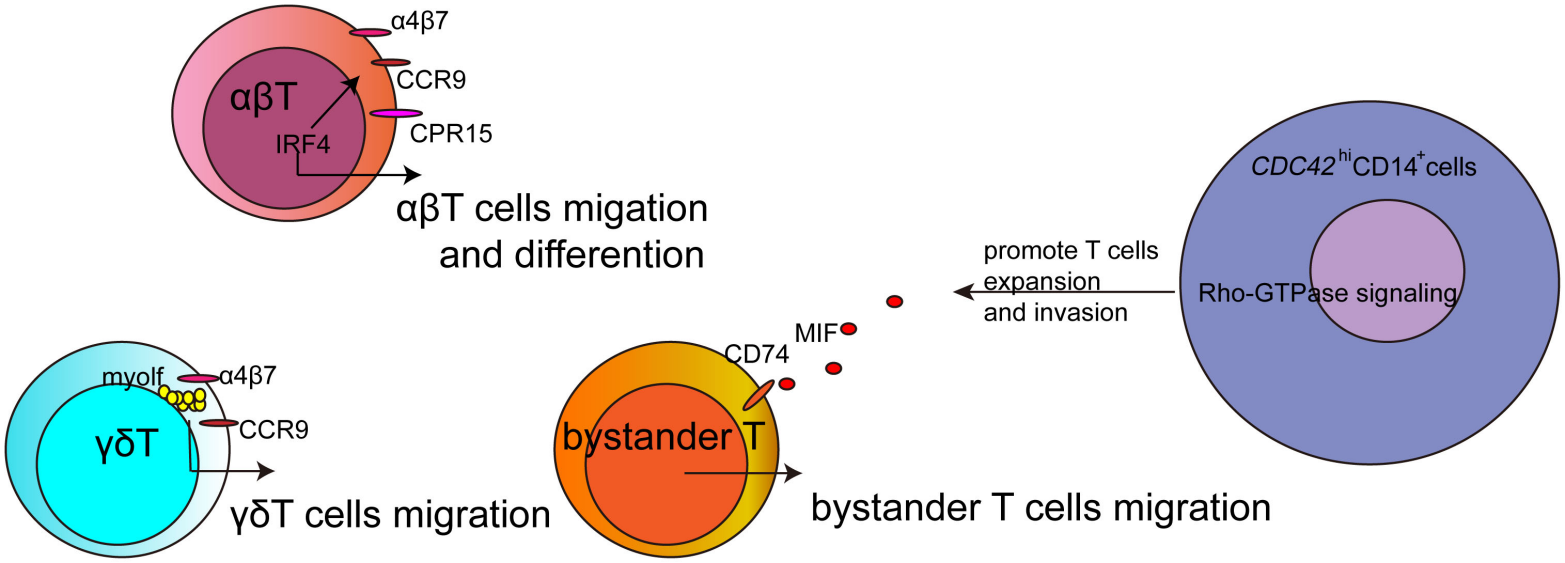
The sketch about the regulatory factors for T cells migration in this Research Topic. Intravital molecular imaging provied powerful technology for studying immune cells migration *in vivo*.

## Author contributions

WL: Funding acquisition, Writing – original draft, Writing – review & editing.
